# Immunosuppression regimen and latitude impact keratinocyte carcinoma risk in U.S. liver transplant recipients

**DOI:** 10.1007/s00403-024-03404-3

**Published:** 2024-09-26

**Authors:** Benjamin E. Rosenthal, Douglas E. Schaubel, James D. Lewis, David J. Margolis, David S. Goldberg, Therese Bittermann

**Affiliations:** 1 Department of Medicine, University of Pennsylvania, Philadelphia, PA USA; 2 Department of Biostatistics, Epidemiology and Informatics, University of Pennsylvania, Philadelphia, PA USA; 3 Division of Gastroenterology, Department of Medicine, University of Pennsylvania, Philadelphia, PA USA; 4 Department of Dermatology, Perelman School of Medicine, University of Pennsylvania, Philadelphia, USA; 5 Division of Digestive Health and Liver Diseases, Department of Medicine, Miller School of Medicine at the University of Miami, Miami, FL USA; 6 Hospital of the University of Pennsylvania, 2 Dulles – Penn Transplant Institute, 3400 Spruce Street, Philadelphia, PA 19104 USA

**Keywords:** Liver transplantation, Immunosuppression, Latitude, Keratinocyte carcinoma

## Abstract

Immunosuppression after solid organ transplantation is associated with an increased risk of keratinocyte carcinoma (KC). Despite its established morbidity, KC risk in liver transplant (LT) recipients is understudied, including the contribution of immunosuppression regimen and latitude. A retrospective cohort of 9,966 adult first LT alone recipients alive with their native allograft at 1-year post-LT without prior KC between 2007 and 2016 were identified using linked data from the Organ Procurement and Transplantation Network and Medicare administrative claims. The primary exposures were immunosuppression regimen and latitude of residence. The primary outcome was incident, *de novo* KC occurring at least 1-year after LT. Adjusted Cox regression analysis stratified by transplant center was used in all analyses. The cohort was 63.4% male, 70.2% White and with median age 61 years (interquartile range, IQR, 54–66) at transplant. Calcineurin inhibitor (CNI) with anti-metabolite combination was independently associated with incident KC when measured as intention-to-treat (adjusted hazard ratio (aHR) 1.21 vs. CNI monotherapy, 95% CI 1.02–1.43, *p* = 0.026), in a time-updating as-treated analysis (aHR 1.61, 95% CI 1.34–1.93; *p* < 0.001) and when measured as cumulative exposure (aHR 1.13 per 6-month increase, 95% CI: 1.02–1.33; *p* = 0.027). More southern latitude of residence was also independently associated with incident KC with an aHR of 1.26 per 5°N decrease towards the Equator (95% CI: 1.08–1.47, *p* = 0.003). We demonstrate independent effects of CNI with antiM immunosuppression regimen and latitude of residence on the risk of post-LT KC, which will better inform screening practices and immunosuppression management.

**Supplementary Information:**

The online version contains supplementary material available at 10.1007/s00403-024-03404-3.

## Introduction

The long-term immunosuppression required to maintain allograft function after solid organ transplantation leads to decreased tumor surveillance and an increased risk of malignancy. Keratinocyte carcinomas (KCs), which include squamous and basal cell carcinomas of the skin, are the most common malignancy in transplant recipients, with a risk that is 10- to 65-fold higher than the general population [1]. In organ transplant recipients, KC are more likely to be aggressive, recurrent and metastatic compared to the non-transplant population, leading to greater morbidity and even mortality [2]. 

Established risk factors for post-transplant KC have included male sex, older age and organ transplanted as a surrogate for intensity of immunosuppression, among others [3, 4]. However, the differential effects of specific immunosuppression regimens are incompletely studied, particularly in liver transplant (LT) recipients. Yet, the liver is the second most frequently transplanted organ with approximately 9,000 performed annually in the U.S [5]. Addressing this knowledge gap is also relevant given the limited lack of specific guidance from major international societies on optimal immunosuppression practices after LT, as well as the significant practice heterogeneity that exists among LT centers in the U.S that our group has previously demonstrated [6–9]. Moreover, no study has quantified the impact of decreasing latitude with post-transplant KC, despite the known association between latitude and skin cancers in non-transplant recipients [10]. Yet, when comparing countries worldwide, the highest incidence of post-transplant non-melanoma skin cancer has been reported in Australia, particularly in subtropical Queensland, which lies close to the Equator [11–13]. Thus, it is likely that a relationship between latitude and post-transplant KC risk also exists in North America.

This study investigates the independent effects of immunosuppression regimen and latitude of residence on the risk of first KC among LT recipients in the U.S. Establishing this evidence would have implications for screening and surveillance, as well as for individualizing post-transplant care.

## Methods

### Data source & study population

This study used data from the Organ Procurement and Transplantation Network (OPTN). The OPTN data system includes data on all donor, wait-listed candidates, and transplant recipients in the US, submitted by the members of the OPTN. The Health Resources and Services Administration, U.S. Department of Health and Human Services provides oversight to the activities of the OPTN contractor. These OPTN data were linked to Medicare insurance billing claims provided by the Centers for Medicare and Medicaid Services [6, 14]. Medicare is the largest single-payer of transplant-related care in the U.S. and the primary payer for 20–25% of LT in the U.S [6, 15]. The study cohort included all adult first liver alone transplant recipients between January 1, 2007 and December 31, 2016, who were actively enrolled in Part A (inpatient) and B (outpatient) Medicare at the time of LT as a result of age ≥ 65 years or disability. Multiorgan and prior transplant recipients were excluded, given inherent differences in immunosuppression. To ensure the adequate capture of immunosuppression regimen, the cohort was limited to patients with ≥ 1 non-corticosteroid immunosuppression prescription fill in the first 90-days after LT [6, 14]. Patients with active Medicare Part A due to age or disability at transplant can receive indefinite coverage for transplant immunosuppression drugs if they have active Part B at the time of the prescription fill [16]. Thus, the study population was not limited to patients with an out-of-pocket Part D prescription plan.

The final study cohort included all patients alive with their native allograft at 1-year post-LT, with no evidence of pre-LT or early (< 1 year) post-LT KC (Figure S1). This latter restriction was applied for several reasons. First, skin cancer screening is recommended annually after transplant, such that there was concern that KCs < 1 year represented pre-existing, rather than *de novo* post-transplant KC cases [17]. Second, there is latency between when a skin cancer appears and when it is treated by a dermatologist, again raising the concern that these early KCs were present pre-LT [18]. Lastly, due to the increased risk of rejection early post-transplant, immunosuppression is often not easily modifiable in the first post-LT year. The greater flexibility in drug choice and intensity beyond this time point renders the question of differential KC risk by regimen of greater interest to the transplant community.

### Exposures and covariates

The primary exposures of interest were immunosuppression regimen and latitude of residence. Induction immunosuppression and maintenance regimen at LT hospital discharge were derived from the OPTN database. Maintenance immunosuppression regimen at all time points beyond LT hospital discharge were obtained using Medicare claims data. These were queried for oral formulations of: calcineurin inhibitors (CNIs; tacrolimus and cyclosporine), anti-metabolites (antiM; mycophenolic acid derivatives and azathioprine), corticosteroids (prednisone and budesonide) and mechanistic target of rapamycin inhibitors (mTORi; sirolimus and everolimus). Common regimens were then defined as in prior studies using this U.S. Medicare cohort: three-drug therapy with CNI + antiM + steroid, two drug therapy with either CNI + antiM or CNI + steroid, CNI monotherapy or mTORi-based therapy [14, 19, 20]. During the first 5-years post-LT, the most frequently used long-term regimens in this study population were CNI monotherapy (∼ 50–70% of recipients) and CNI + antiM (∼ 10–20%), with other regimens representing < 10% each. Among patients receiving CNI, tacrolimus was used in > 90% of patients, while mycophenolic acid derivatives were used in > 90% of those receiving antiM therapy. As per the available clinical trial data, the most commonly used long-term mTOR inhibitor-based regimens in LT recipients are mTOR inhibitor monotherapy or combination therapy with a dose-reduced CNI, though additional regimens may be used [21]. Antibody-based induction immunosuppression included anti-CD25 monoclonal antibody induction (i.e., interleukin-2 receptor antagonists) and polyclonal antibody induction (e.g., anti-thymocyte globulin) [22]. Latitude of residence was computed by obtaining the geographic coordinates of the population-weighted centroid of patients’ home zip code at LT, as listed in the Medicare Master Beneficiary Summary File.

Additional covariates were obtained at LT using the OPTN database. These included sex, age, race/ethnicity, primary liver disease, history of hepatocellular carcinoma, history of diabetes, Model for End-Stage Liver Disease (MELD) and receipt of hemodialysis in the week prior to LT. In addition, history of acute rejection during the first post-LT year was captured as a binary variable. Of note, OPTN does not record specific dates of acute rejection episodes but rather as binary (Y/N) variables at the following time points: during the LT admission and at 6- and 12-months post-LT.

### Outcomes

The primary outcome of the study was incident, *de novo* KC occurring at least 1-year after LT. This outcome was derived using a validated algorithm that includes both International Classification of Diseases (ICD) 9/10 codes and Common Procedural Terminology (CPT) codes (Table S1) [23]. Secondary outcomes included: number of total KC procedures during follow-up, procedure type(s) and KC type(s).

### Statistical analysis

Cell sizes < 10 were not shown as per Medicare data rules. To assess differences between patients with and without *de novo* KC, Chi-square and Kruskal-Wallis tests were used to compare categorical and continuous variables, respectively. The KC incidence rate (IR) starting at 1 year from LT was calculated per 1,000 person-years (PY) of observation. Secondary outcomes were described.

In a first regression analysis, covariate-adjusted Cox proportional hazards analysis evaluated the association of immunosuppression and latitude of residence on post-LT KC risk. For this analysis, immunosuppression regimen measured at 1-year from LT was evaluated in an intention-to-treat (ITT) manner. Time zero was set at 1-year from LT and patients were followed until first KC occurrence (failure event) or retransplantation or death (censoring events), whichever occurred first. Latitude of residence was evaluated continuously in increments of 5°N towards the Equator and as a categorical variable (≥ 40°N [reference], 35–39°N, 30–34°N and < 30°N) in a sensitivity analysis. All multivariable models adjusted for the aforementioned covariates. As a secondary analysis, the interaction of immunosuppression regimen at 1-year and latitude of residence was investigated. All models were stratified by transplant center to account for clustering of patient characteristics including immunosuppression practices. Note that, unlike a frailty (mixed model) approach, stratification does not assume that center is independent of the remaining predictors. Additionally, each Cox regression analysis accommodated the competing risk of death by modeling the cause-specific KC hazard (i.e., the KC rate while patients are actually at-risk (alive)) [24]. 

In a second ‘as treated’ analysis, immunosuppression regimen was evaluated as a time-updating exposure. For computational convenience, the regimen that patients received at the start of each month was used. Correspondingly, the time to event was also recorded in months. We used Cox regression stratified by center and each model included immunosuppression maintenance regimen, latitude of residence (continuous), and the same adjustment covariates included in the ITT models. To account for a potential lag between immunosuppression and incident skin cancer, multiple lag times were simulated (0-, 3-, 6-, 9- and 12-months). For example, for a 6-month lag time, exposure at month 18 is based on the immunosuppression regimen at 12 months post-LT. Lastly, we fit Cox models that assessed cumulative regimen exposure (per 6-month time intervals) and, separately, Cox models with cumulative exposure to individual immunosuppression drug classes, rather than regimen (for non-mTOR-inhibitor regimens).

This study was approved as exempt by the Institutional Review Board of the University of Pennsylvania. All analyses were conducted using Stata v18 (College Station, TX) and SAS (Cary, NC).

## Results

### Overall characteristics of the study population

The final study cohort included 9,966 patients who underwent LT between 1/1/2007-12/31/2016 and were alive with their native allograft at 1-year post-LT (Figure S1). All 50 U.S. States were represented in the sample. Baseline characteristics are summarized in Table 1. The cohort was 63.4% male, 70.2% White, 8.6% Black, 15.5% Hispanic, and 4.3% Asian with median age 61 years (IQR: 54–66). Primary indications for LT included hepatitis C virus (36.5%), alcohol-associated liver disease (ALD; 21.7%) and metabolic dysfunction-associated steatotic liver disease (MASLD; 14.2%). There were 3,052 patients (30.6%) with HCC and 3,137 (31.7%) with diabetes prior to LT. Median MELD score at transplant was 18 (IQR 12, 26). The majority of patients did not receive antibody-based induction therapy (71.3%). Maintenance regimen at LT hospital discharge was 64.6% CNI + antiM + steroid, 17.4% CNI + antiM, 9.7% CNI + steroid and 3.8% CNI alone. Overall, 10.3% experienced acute rejection during the first post-LT year. Figure S2 displays the distribution of latitude of residence for the study cohort.

**Table 1:** Baseline characteristics of the study cohort (*n* = 9,966)

	Overall cohort (*n* = 9,966)
Sex, *N*(%)	
Male Female	6,319 (63.4) 3,647 (36.6)
Age at transplant (years), median (IQR)	61 (54, 66)
Race/ethnicity, *N*(%)	
Non-Hispanic White Non-Hispanic Black Hispanic Asian Other	6,994 (70.2) 856 (8.6) 1,544 (15.5) 427 (4.3) 145 (1.5)
Liver disease, *N*(%)	
Alcohol MASLD Hepatitis C virus Hepatitis B virus Autoimmune hepatitis Primary biliary cholangitis Primary sclerosing cholangitis Other	2,164 (21.7) 1,415 (14.2) 3,639 (36.5) 312 (3.1) 267 (2.7) 372 (3.7) 350 (3.5) 1,447 (14.5)
Hepatocellular carcinoma at LT, *N*(%)	3,052 (30.6)
Diabetes prior to LT, *N*(%)	3,137 (31.7)
Native MELD score at LT, median (IQR)	18 (12, 26)
Induction immunosuppression at LT, *N*(%)	
No antibody-based induction Anti-CD25 monoclonal antibody induction Polyclonal antibody induction	7,110 (71.3) 1,783 (17.9) 1,073 (10.8)
Immunosuppression at discharge post LT, *N*(%)	
CNI + antiM + steroid CNI + antiM CNI + steroid CNI alone Other/unknown	6,427 (64.6) 1,732 (17.4) 966 (9.7) 378 (3.8) 441 (4.4)
Acute rejection during first post-LT year, N(%)	1,023 (10.3)

Abbreviations: AntiM – antimetabolite; CNI – calcineurin inhibitor; MASLD – metabolic dysfunction-associated steatotic liver disease; MELD – Model for End-stage Liver Disease

### Incidence and morbidity of post-LT KC

**Fig. 1:**
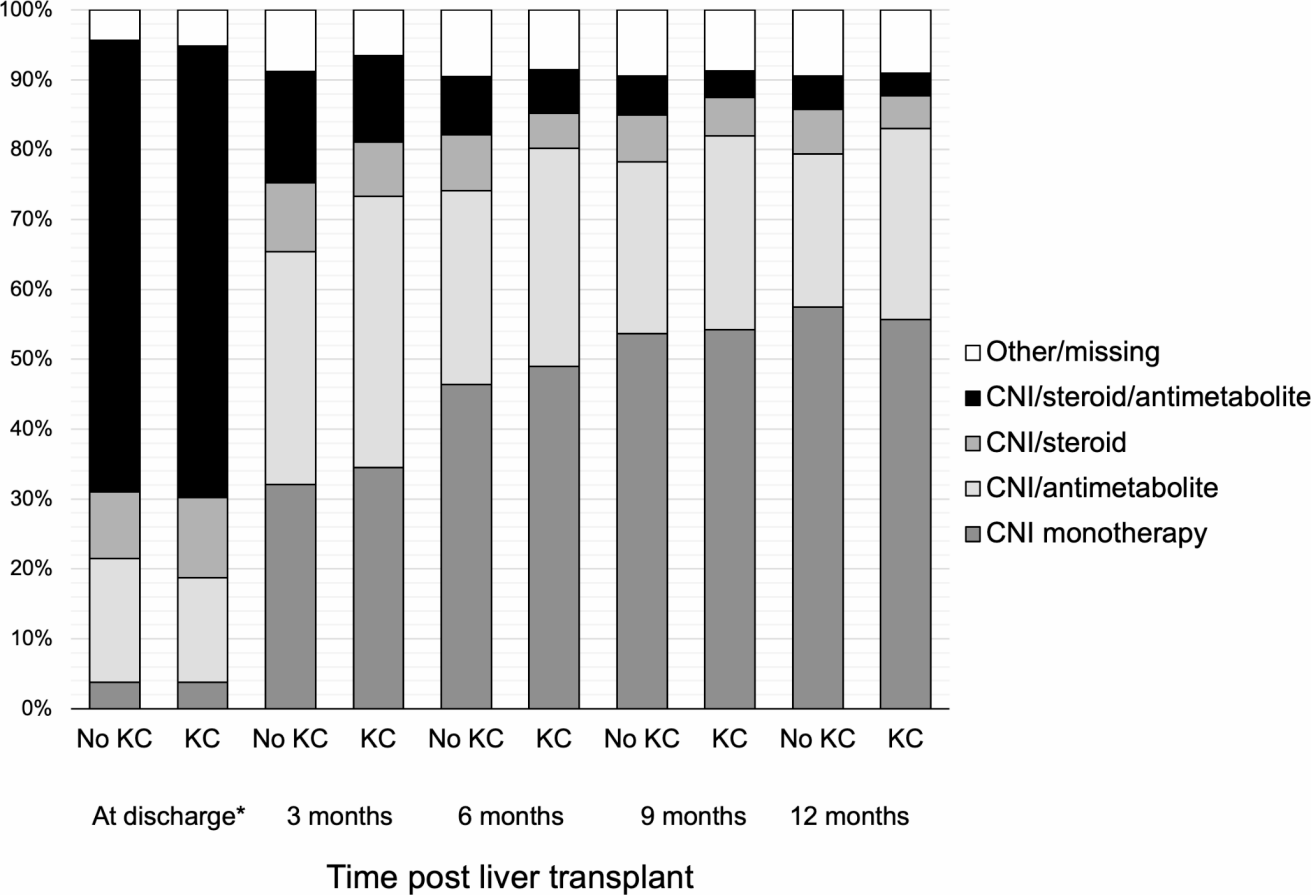
Immunosuppression regimen at discharge and 3-month intervals up to one year post liver transplant obtained from Medicare claims data. * Patients who would subsequently develop keratinocyte carcinoma (KC) are compared to those who did not *Discharge immunosuppression regimen was obtained from the Organ Procurement and Transplantation Network database

The 9,966 patients in the study cohort contributed to a total of 38,502 person-years during follow-up. In total, 1,033 (10.4%) incident cases of *de novo* post-LT KC were identified, resulting in an incidence rate (IR) of 26.8 (95% CI 25.2–28.5) per 1,000 PY. The highest IRs of KC were in recipients aged ≥ 70 years at LT (43.2 per 1,000 PY, *p* < 0.001), followed by patients of non-Hispanic White race (37.5 per 1,000 PY, *p* < 0.001; Table 2). Men had significantly higher KC IR than women: 31.2 vs. 19.7 per 1,000 PY (*p* < 0.001). The IR of KC was lower for patients with viral hepatitis (21.8 per 1,000 PY for hepatitis C and 17.4 per 1,000 PY for hepatitis B (*p* < 0.001 and *p* = 0.007 versus ALD as the reference). With more southern latitude of residence, the KC IR increased: 21.6 per 1,000 PY for latitude ≥ 40°N, 28.5 per 1,000 PY for latitude 35–39°N, 28.4 per 1,000 PY for latitude 30–34°N and 30.9 per 1,000 PY for latitude < 30°N (*p* = 0.001, *p* = 0.003 and *p* = 0.002 versus latitude ≥ 40°N as reference). There were no differences in induction therapy type (*p* = 0.453), maintenance regimen at LT discharge (*p* = 0.063) or acute rejection during the first post-LT year (*p* = 0.917) between patients with and without post-LT KC (Table S2). Within the first year after liver transplant, the majority of KC and non-KC patients transitioned to CNI monotherapy as seen in Fig. 1.

**Table 2:** Incidence rates of keratinocyte carcinoma (KC) post liver transplant (LT) by sex, age at transplant, race, liver disease and latitude category between 1/1/2007-12/31/2016

	KC incidence rate per 1,000 PY, (95% CI)	*p*-value
Sex, *N*(%)		< 0.001
Male Female	31.2 (29.0-33.5) 19.7 (17.6–22.1)	
Age at transplant (years)		
<50 50–59 60–69 ≥70	8.9 (6.8–11.7) 21.3 (19.0-23.8) 35.0 (32.3–38.0) 43.2 (36.1–51.7)	Reference < 0.001 < 0.001 < 0.001
Race/ethnicity, *N*(%)		
Non-Hispanic White Non-Hispanic Black Hispanic Asian Other	37.5 (35.2–39.9) 1.2 (0.4–3.1) 6.0 (4.4–8.2) 4.0 (2.0-8.1) 11.2 (5.0-24.9)	Reference < 0.001 < 0.001 < 0.001 < 0.001
Liver disease, *N*(%)		
Alcohol MASLD Hepatitis C virus Hepatitis B virus Autoimmune hepatitis Primary biliary cholangitis Primary sclerosing cholangitis Other	29.7 (26.2–33.7) 30.1 (25.7–35.4) 21.8 (19.5–24.4) 17.4 (11.8–25.6) 24.4 (16.6–35.8) 21.6 (15.4–30.2) 34.5 (25.9–45.9) 34.3 (29.9–39.3)	Reference 0.871 < 0.001 0.007 0.340 0.075 0.347 0.132
Latitude of residence, *N*(%)		
≥40 °*N* 35–39 °*N* 30–34 °*N* <30 °*N*	21.6 (19.0-24.4) 28.5 (25.4–32.0) 28.4 (25.0-32.2) 30.9 (25.8–37.0)	Reference 0.001 0.003 0.002

Abbreviations: MASLD – metabolic dysfunction-associated steatotic liver disease

Among the 1,033 patients with KC in the cohort, a total of 2,462 KC-associated procedures were performed during follow-up. While the majority (53.8%) of patients underwent one procedure, 18.9% underwent 2 procedures, 16.7% underwent 3–4, and 10.7% underwent ≥ 5 for KC during follow-up (Table 3). Men were more likely to undergo more procedures compared to women (*p* = 0.027). Women were more likely to experience only basal cell carcinomas (36.9% vs. 28.2%) and less likely to experience only squamous cell carcinomas (31.4% vs. 38.9%; *p* = 0.001). They were also less likely to undergo Mohs surgery compared to men (51.2% vs. 61.8%; *p* = 0.006). There were no other significant differences in the number or type of procedure(s) or the type of KC(s) among other variables including age, liver diagnosis, latitude category or maintenance immunosuppression regimen at 1-year post-LT (all *p* > 0.05; data not shown).

**Table 3:** Morbidity of KC among 1,033 LT recipients who developed *de novo* NMSC post LT between 1/1/2007 and 12/31/2006 stratified by sex

	Overall (*N* = 1,033)	Men (*N* = 746)	Women (*N* = 287)	*p*-value
Total procedures, *N*(%)				0.027
1 2 3–4 ≥5	556 (53.8) 195 (18.9) 172 (16.7) 110 (10.7)	392 (52.6) 132 (17.7) 133 (17.8) 89 (11.9)	164 (57.1) 63 (22.0) 39 (13.6) 21 (7.3)	
Type(s) of KC, *N*(%)				0.001
Only squamous cell Only basal cell Both squamous and basal Other/unspecified	380 (36.8) 316 (30.6) 174 (16.8) 163 (15.8)	290 (38.9) 210 (28.2) 139 (18.6) 107 (14.3)	90 (31.4) 106 (36.9) 35 (12.2) 56 (19.5)	
Procedure type				0.006
Any Mohs Any excision, no Mohs Destruction only	608 (58.9) 228 (22.1) 197 (19.1)	461 (61.8) 157 (21.1) 128 (17.2)	147 (51.2) 71 (24.7) 69 (24.0)	

### Association of immunosuppression regimen and latitude on post-LT KC risk

In the covariate-adjusted ITT analysis, a higher risk of KC was observed with CNI + antiM at 1-year compared to CNI monotherapy: adjusted HR (aHR) 1.21 (95% CI: 1.02–1.43; *p* = 0.026; Table 4). The risk of post-LT KC increased with more southern latitude of residence with an aHR of 1.26 (95% CI: 1.08–1.47; *p* = 0.003) per 5°N towards the Equator. In the sensitivity analysis that used categorical latitude, the following aHRs were obtained versus ≥ 40°N as the reference: 1.37 (95% CI; 1.00-1.87; *p* = 0.048) for 35–39°N, 1.69 (95% CI: 1.14–2.51; *p* = 0.009) for 30–34°N and 2.33 (95% CI: 1.48–3.67; *p* < 0.001) for < 30°N (Table S3). On multivariable analysis, there was no interaction between maintenance regimen at 1-year post-LT and latitude of residence (*p* = 0.123).

**Table 4:** Factors associated with post-LT KC in unadjusted and adjusted Cox regression analyses

	Adjusted hazard ratio* (95% CI)	*p*-value
Female sex	0.65 (0.56–0.76)	< 0.001
Age at LT per 5-year increase	1.32 (1.25–1.38)	< 0.001
Maintenance immunosuppression		
CNI + antiM + steroid CNI + antiM CNI + steroid CNI alone Other/unknown	Reference 1.21 (1.02–1.43) 0.91 (0.65–1.25) 0.99 (0.67–1.46) 0.85 (0.67–1.10)	Reference 0.026 0.548 0.968 0.233
Latitude of residence, per 5°N toward equator	1.26 (1.08–1.47)	0.003
Race/ethnicity, *N*(%)		
Non-Hispanic White Non-Hispanic Black Hispanic Asian Other	Reference 0.04 (0.02–0.11) 0.16 (0.11–0.23) 0.08 (0.04–0.17) 0.43 (0.19–0.97)	Reference < 0.001 < 0.001 < 0.001 0.043
Liver disease, *N*(%)		
Alcohol MASLD Hepatitis C virus Hepatitis B virus Autoimmune hepatitis Primary biliary cholangitis Primary sclerosing cholangitis Other	Reference 0.70 (0.55–0.88) 0.81 (0.67–0.98) 1.08 (0.68–1.71) 1.18 (0.75–1.86) 0.71 (0.47–1.08) 0.96 (0.66–1.40) 1.08 (0.88–1.34)	Reference 0.003 0.033 0.743 0.470 0.110 0.835 0.449
Induction immunosuppression at LT, *N*(%)		
No antibody-based induction Anti-CD25 monoclonal antibody induction Polyclonal antibody induction	Reference 1.08 (0.87–1.34) 0.86 (0.60–1.23)	Reference 0.474 0.409
Rejection at one year	1.08 (0.86–1.36)	0.500
Dialysis at transplant	1.02 (0.72–1.45)	0.892
MELD at transplant per 5 points	1.00 (0.95–1.05)	0.947
Hepatocellular carcinoma	1.00 (0.84–1.20)	0.982

*Multivariable model adjusted for the following covariates: type of immunosuppression, latitude, sex, age, race, diagnosis, diabetes, MELD score, induction therapy, rejection at one year, presence of hepatocellular carcinoma and use of hemodialysis at transplant

Abbreviations: AntiM – antimetabolite; CNI – calcineurin inhibitor; MASLD – metabolic dysfunction-associated steatotic liver disease; MELD – Model for End-stage Liver Disease

Increasing age at LT was associated with an increase in the hazard of KC: aHR 1.32 per 5-year increase (95% CI: 1.25–1.38; *p* < 0.001). Factors independently associated with a lower risk of *de novo* post-LT KC included: female sex (aHR 0.65, 95% CI 0.56–0.76; *p* < 0.001), Non-Hispanic Black race (aHR 0.04, 95% CI: 0.02–0.11), Hispanic ethnicity (aHR 0.16, 95% CI: 0.11–0.23) and Asian race (aHR 0.08, 95% CI: 0.04–0.17; *p* < 0.001 race/ethnicity overall). Patients with MASLD or hepatitis C virus had lower risk of KC compared to those with ALD: aHR 0.70 (95% CI 0.55–0.88, *p* = 0.003) and aHR 0.81 (0.67–0.98, *p* = 0.033), respectively. Neither induction therapy nor history of acute rejection were associated with KC risk in the final multivariable model (*p* = 0.500 for both).

In the adjusted ‘as treated’ analysis using immunosuppression regimen as a time-dependent exposure, a higher risk of KC was again observed with CNI + antiM compared to CNI monotherapy: aHR 1.61 (95% CI: 1.35–1.93; *p* < 0.001). To account for a potential lag between immunosuppressant exposure and KC risk, lag time models showed that the increased KC hazard of CNI + anitM decreased but did not disappear with increasing lag time and remained significant with a lag time of 12 months: aHR 1.35 (95% CI 1.12–1.61, *p* = 0.001; Table S4). In contrast, mTOR based immunosuppression regimens had a non-significant trend toward increased KC compared to CNI monotherapy: HR 1.25 (95% CI: 0.98–1.59, *p* = 0.08), however this effect diminished in size increasing lag time (*p* > 0.05; Table S4).

We also fit our adjusted Cox models to cumulative immunosuppressant exposure, with results shown in Table S5. This demonstrated increased skin cancer incidence risk with each 6-months of immunosuppressant exposure. The increase was strongest for CNI + antiM: aHR 1.18.

(95% CI 1.02,1.37, *p* = 0.027) per 6-month increase in cumulative exposure. We then evaluated the association of cumulative exposure to individual immunosuppression drug types. Cumulative exposure to CNI was the primary driver of the increase in KC risk seen with CNI-based regimens such as CNI + antiM: with each 6-months of additional exposure being associated with a 19% increase in the hazard of KC (aHR: 1.19, 95% CI 1.04–1.37, *p* = 0.012; Table S6).

## Discussion

Our study aimed to elucidate the independent effects of immunosuppression regimen and latitude of residence on the risk of first KC among LT recipients in the U.S. We find that immunosuppression with a CNI combined with an antiM is associated with a higher risk of incident KC, a finding that was robust to using three different analytic approaches: in the ITT model, in a time-updating ‘as-treated’ analysis that simulated multiple lag times, and when estimating cumulative exposure over time. Since CNI + antiM is the second most frequently employed long-term immunosuppression strategy in LT recipients, with 15–20% of patients receiving this regimen for the first 3 years post-LT, this finding has key implications with respect to KC screening and surveillance [19]. Moreover, this represents a novel, potentially modifiable risk factor that should be considered when determining the optimal immunosuppression strategy for patients at high risk of first or subsequent post-LT KC. This study also identified decreasing latitude towards the Equator, as a surrogate for UV exposure, as a significant risk factor for incident post-LT KC. Patients who reside at more southern latitudes should therefore be considered for increased KC screening and surveillance, and potentially also from immunosuppression modification (if feasible) to reduce the risk of *de novo* KC post-LT and its associated morbidity.

Our analyses suggest that the average effect of individual regimen components is largely additive (e.g., aHR of 1.22 per 6-month cumulative exposure to CNI + antiM and aHRs of 1.19 and 1.02 for that to CNI and antiM, respectively). This finding additionally highlights the inherent nuances of immunosuppression-related cancer risk: while antiM are often implicated in the development of KC non-transplant settings, the CNI component of CNI + antiM regimens was still the primary driver of KC risk in this cohort. We hypothesize this may be due to antiM dosing in LT recipients being generally lower than for other indications (e.g., inflammatory bowel disease). It is also important to recognize that these findings represent the average effects of immunosuppression on KC risk in the cohort and that there is likely substantial variation between patients. With respect to individual drug exposures, we previously showed in a prior publication describing treatment patterns in this U.S. Medicare cohort that > 90% of patients receiving CNI were treated with tacrolimus during the first 5-years post-LT, and > 90% of those receiving antiM were on mycophenolic acid derivatives [19]. Studies of non-liver solid organ transplants have suggested that azathioprine is associated with a higher risk of post-transplant skin cancers than mycophenolic acid derivatives [25–27]. Patients in this cohort receiving dual treatment with CNI and an antiM, which primarily consisted of a mycophenolic acid derivative, experienced a 20% higher risk of KC than with CNI alone. While international society guidelines do not favor the use of one antiM over another in LT recipients [7–9], KC risk was likely higher among the small subset of LT recipients treated with azathioprine given these prior data.

Beyond these findings, we find that patients with MASLD or hepatitis C virus have lower KC risk than patients with ALD. We hypothesize this is due to unmeasured confounding, potentially related to UV exposure or other factors (e.g., tobacco use), given that it is unlikely that liver disease itself alters the risk of post-LT KC. Similar to other investigators, we identified male sex and increasing age as additional important predictors of KC after transplant [3]. The high incidence and morbidity associated with post-LT KC also underscore the importance of individualized post-transplant care strategies. In this cohort, nearly half received multiple KC procedures and nearly 60% of patients underwent Mohs surgery, which can be disfiguring and may lead to reduced health-related quality of life [28, 29]. 

The results from this study add to the available literature on the relationship between increased KC risk and combination immunosuppression with CNI and antiM, particularly using azathioprine, and increased KC risk that originates from other solid organ transplant populations around the world [25, 30, 31]. Several prior studies have also suggested an improvement in KC risk when using mTOR inhibitor-based immunosuppression regimens, including in LT recipients [32–37]. While this finding was not observed in our cohort, this immunosuppression approach could be considered for LT recipients at high risk of post-LT KC. In multiple European countries, skin cancer incidence after organ transplantation has decreased over time, owing to key changes in immunosuppression practices that were consistent across centers, as well as other relevant nationwide programs such as skin cancer screening efforts and sun exposure recommendations [38–40]. However, such widespread practice changes have not occurred to the same degree in the U.S., where substantial variation in immunosuppression management and barriers to skin cancer screening exist [6, 22, 41–43]. 

Several limitations should be acknowledged. The findings obtained among Medicare beneficiaries may not be generalizable to the broader LT population (e.g., due to their increased age and/or other characteristics). Latitude is an imperfect proxy for UV exposure and future prospective studies are needed to better assess factors such as skin photosensitivity and prior sun exposure. There is undoubtedly a delay between the date the KC occurs and when it is first detected (either by patient or physician). As the former is not known, we chose to model various lag-times in our analyses, which showed persistence of the relationship between CNI + antiM and incident KC. The race/ethnicity classifications used do not reflect the granularity of the Fitzpatrick skin phenotype scale, a tool that has been demonstrated to be an independent predictor of post-transplant skin cancer [44]. There is possibility of ascertainment bias in that some patients may have been more diligently screened than others based on their location of residence and race/ethnicity, among other reasons. Lastly, we did not have access to CNI trough levels, which are likely to be variable in the cohort, yet al.so associated with KC.

Immunosuppression regimen and latitude of residence are associated with post-LT KC among Medicare-enrolled LT recipients. Our findings underscore the need for individualized post-transplant care strategies, emphasizing the potential benefits of minimizing exposure to combined CNIs and anti-metabolites and considering geographical variations in UV exposure. Further research is warranted to validate these findings and explore alternative immunosuppressive regimens and sun protection strategies to mitigate the risk of post-LT KC in this high-risk population.

## Electronic supplementary material

Below is the link to the electronic supplementary material.

Supplementary Material 1

Supplementary Material 2

Supplementary Material 3

Supplementary Material 4

## Data Availability

The data used in this study are publicly available via a data use agreement from the Centers for Medicare and Medicaid Services and the Organ Procurement and Transplantation Network. As per the stipulations of the data use agreement for the current study, these data cannot be shared to other entities. However, the investigators are able to share any elements of data acquisition and research methods with other investigators upon request.

## Abbreviations

ALD: Alcohol-associated Liver Disease
antiM: Anti-metabolite
CI: Confidence Interval
CNI: Calcineurin Inhibitor
HCC: Hepatocellular Carcinoma
HR: Hazard Ratio
ICD: International Classification of Diseases
IQR: Interquartile Range
IR: Incidence Rate
ITT: Intention-to-treat
KC: Keratinocyte Carcinoma
LT: Liver Transplantation
MASLD: Metabolic Dysfunction-Associated Steatotic Liver Disease
MELD: Model for End-Stage Liver Disease
mTORi: Mechanistic Target of Rapamycin Inhibitor
OPTN: Organ Procurement and Transplantation Network
PY: Person-Years
