# Supplementary figures and images for: Immunosuppression regimen and latitude impact keratinocyte carcinoma risk in U.S. liver transplant recipients
Source: Arch Dermatol Res. 2024 Sep 26;316(9):641. doi: 10.1007/s00403-024-03404-3 (PMC11427564; doi:10.1007/s00403-024-03404-3)

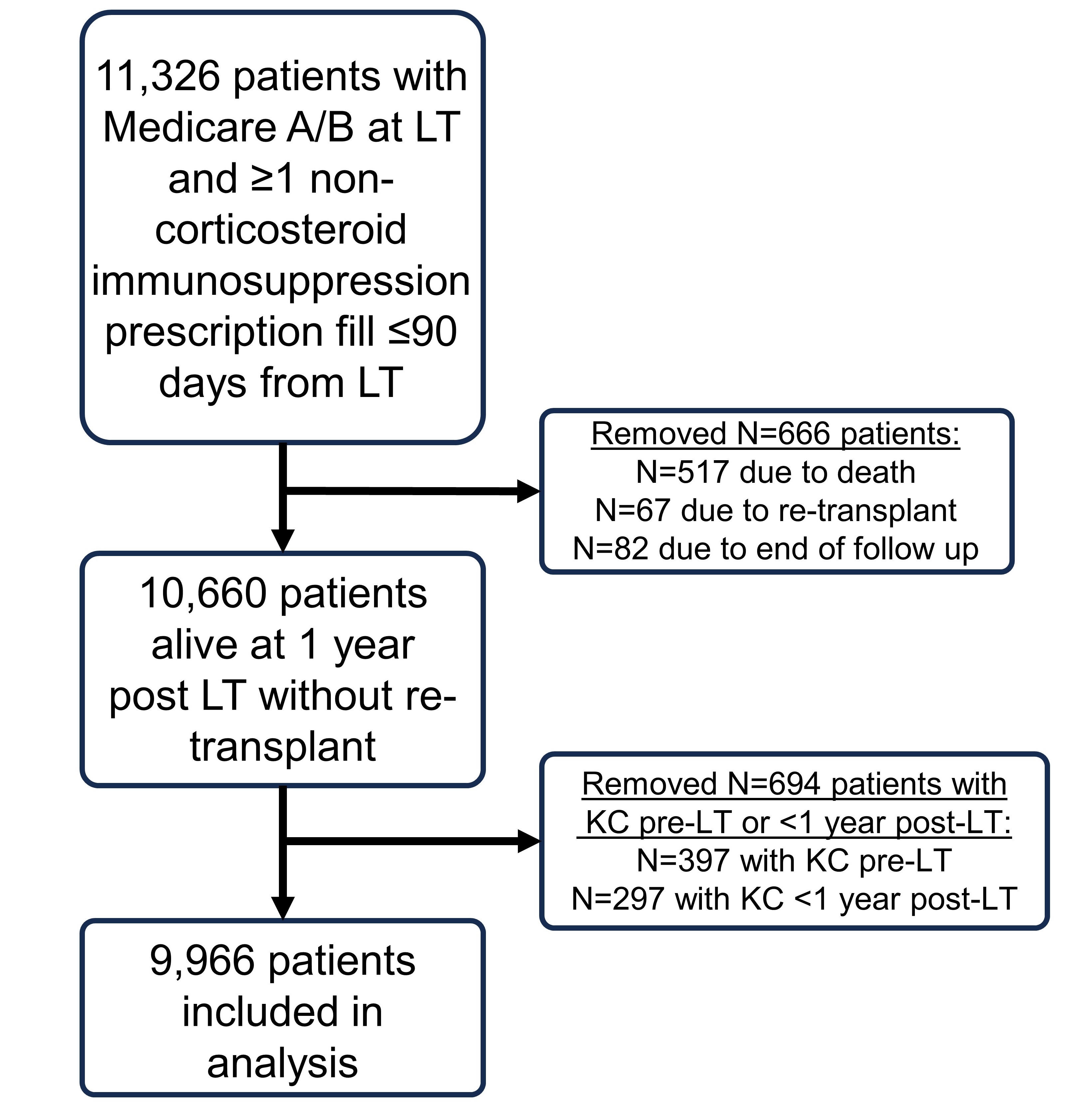

Supplement: Supplementary file 1 — Supplementary Material 1 [file 403_2024_3404_MOESM1_ESM.jpg]

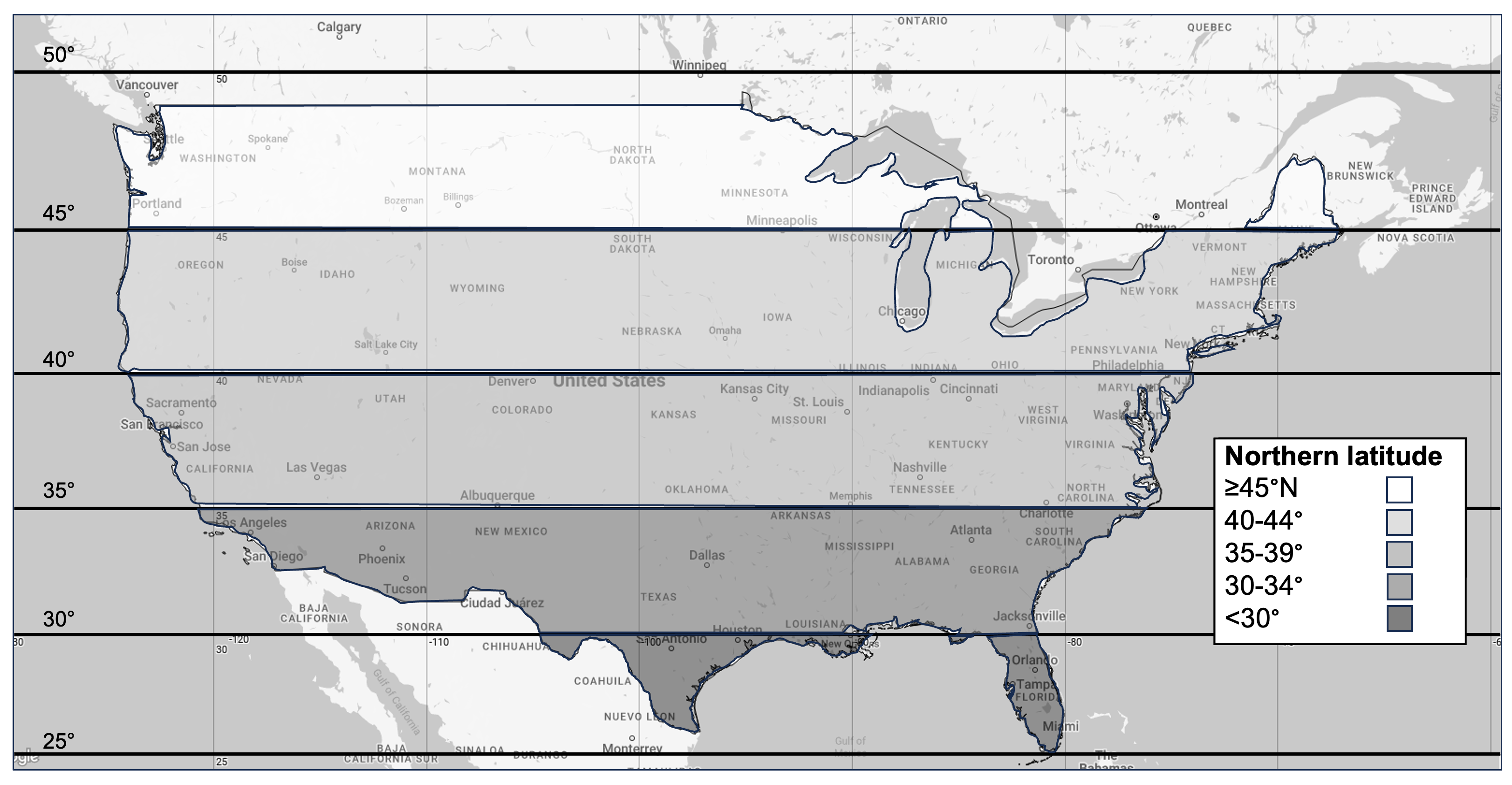

Supplement: Supplementary file 2 — Supplementary Material 2 [file 403_2024_3404_MOESM2_ESM.png]

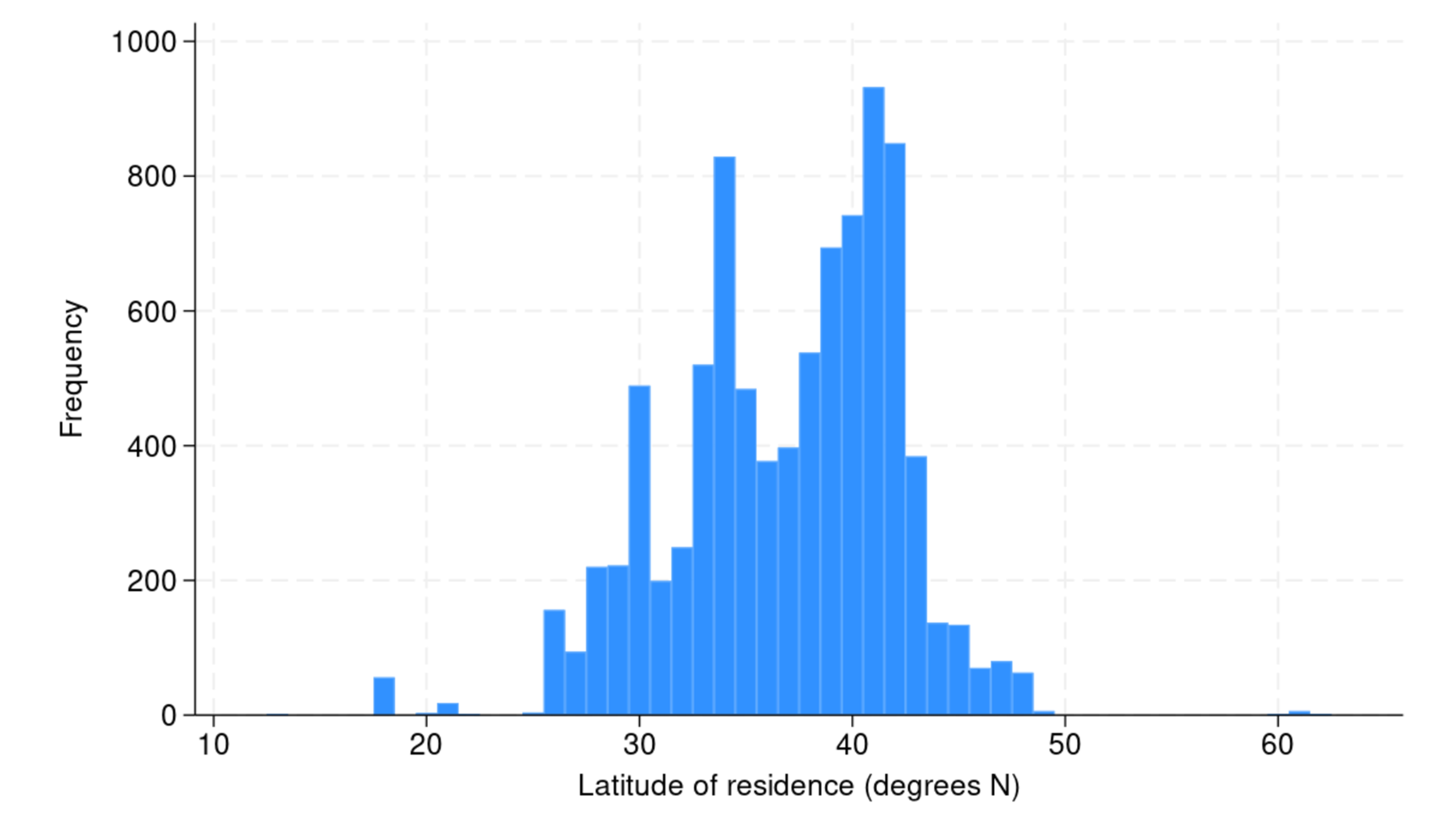

Supplement: Supplementary file 3 — Supplementary Material 3 [file 403_2024_3404_MOESM3_ESM.jpg]
