# Supplementary material for: Immunosuppression regimen and latitude impact keratinocyte carcinoma risk in U.S. liver transplant recipients
Source: Arch Dermatol Res. 2024 Sep 26;316(9):641. doi: 10.1007/s00403-024-03404-3 (PMC11427564; doi:10.1007/s00403-024-03404-3)
Supplement: Supplementary file 4 — Supplementary Material 4 [file 403_2024_3404_MOESM4_ESM.docx]

**Table S1**: Algorithms used to identify various outcomes in the Medicare-UNOS dataset

|  | **Medicare**  **file** | **CPT code** | **ICD-9 codes; dates** | **ICD-10 codes** |
| --- | --- | --- | --- | --- |
| **KC procedure** | Outpatient, carrier | 11600-11606  11620-11626  17260-17266  17270-17276  17280-17286  17311  17313 | 1/1/2007-10/31/2011:  173.0-173.9  After 11/1/2011:  173.01-173.91  173.02-173.92  173.09-173.99  232.0-232.9 | C44.0-44.9 |
|  | **Medicare file** | **E&M code** | **ICD-9 codes; dates** | **ICD-10 codes** |
| **KC diagnosis** | Outpatient, carrier | New outpatient E&M:  99201-99205  Established E&M:  99211-99215  with Medicare  specialty code  07 (dermatology) | 1/1/2007-10/31/2011:  173.0-173.9  After 11/1/2011:  173.01-173.91  173.02-173.92  173.09-173.99  232.0-232.9 | C44.0-44.9 |

**Table S2**: Comparison of early immunosuppression and rejection between patients with keratinocyte carcinoma (KC) and without KC

|  | KC  (n=1,033) | No KC (n=8,933) | p-value |
| --- | --- | --- | --- |
| Induction immunosuppression at LT, N(%)  None/steroid-only  Non-depleting antibody induction  Depleting antibody induction | 754 (73.0)  176 (17.0)  103 (10.0) | 6356 (71.2)  1,607 (18.0)  970 (10.9) | 0.453 |
| Immunosuppression at LT discharge, N(%)  CNI + antiM + steroid  CNI + antiM  CNI + steroid  CNI alone  Other/unknown | 665 (64.6)  154 (15.0)  118 (11.5)  39 (3.8)  53 (5.2) | 5,762 (64.6)  1,578 (17.7)  848 (9.5)  339 (3.8)  388 (4.4) | 0.063 |
| Acute rejection during first post-LT year, N(%) | 107 (10.4) | 916 (10.4) | 0.917 |

**Table S3**: Factors associated with post-LT KC in unadjusted and adjusted Cox regression analyses

|  | **Adjusted hazard ratio***  **(95% CI)** | **p-value** |
| --- | --- | --- |
| Female sex | 0.65 (0.55-0.76) | <0.001 |
| Age at LT per 5-year increase | 1.31 (1.25-1.38) | <0.001 |
| Maintenance immunosuppression  CNI + antiM + steroid  CNI + antiM  CNI + steroid  CNI alone  Other/unknown | Reference  1.21 (1.02-1.43)  0.90 (0.65-1.25)  1.00 (0.68-1.48)  0.86 (0.67-1.10) | 0.025  0.541  0.982  0.235 |
| Latitude of residence  ≥40 °N  35-39 °N  30-34 °N  <30 °N | Reference  1.37 (1.00-1.87)  1.69 (1.14-2.51)  2.33 (1.48-3.67) | 0.048  0.009  <0.001 |
| Race/ethnicity  Non-Hispanic White  Non-Hispanic Black  Hispanic  Asian  Other | Reference  0.04 (0.12-0.11)  0.16 (0.11-0.23)  0.08 (0.04-0.17)  0.43 (0.19-0.98) | Reference  <0.001  <0.001  <0.001  0.044 |
| Liver disease  Alcohol  MASLD  Hepatitis C virus  Hepatitis B virus  Autoimmune hepatitis  Primary biliary cholangitis  Primary sclerosing cholangitis  Other | Reference  0.70 (0.55-0.88)  0.81 (0.67-0.98)  1.07 (0.68-1.70)  1.18 (0.76-1.87)  0.71 (0.47-1.08)  0.96 (0.66-1.41)  1.08 (0.88-1.34) | Reference  0.003  0.032  0.760  0.453  0.111  0.850  0.459 |
| Induction immunosuppression at LT  None/steroid-only  Non-depleting antibody induction  Depleting antibody induction | Reference  1.08 (0.87-1.34)  0.87 (0.61-1.25) | Reference  0.476  0.461 |
| Rejection at one year | 1.09 (0.86-1.37) | 0.480 |
| Dialysis at transplant | 1.01 (0.72-1.44) | 0.936 |
| MELD at transplant per 5 points | 1.00 (0.95-1.05) | 0.989 |
| Hepatocellular carcinoma | 0.99 (0.83-1.19) | 0.953 |

*Multivariable model adjusted for the following covariates: type of immunosuppression, latitude category, sex, age, race, diagnosis, diabetes, MELD score, induction therapy, rejection at one year, presence of hepatocellular carcinoma and use of hemodialysis at transplant.

Abbreviations: AntiM – antimetabolite; CNI – calcineurin inhibitor; MASLD – metabolic dysfunction-associated steatotic liver disease; MELD – Model for End-stage Liver Disease

**Table S4**: Covariate-adjusted hazard ratios for post-LT KC using 0, 3, 6, 9 and 12-month lag times.

| **Lag = 12 months** | p-value | **0.001** | 0.41 | 0.11 | 0.19 |
| --- | --- | --- | --- | --- | --- |
|  | HR  (95% CI) | **1.35  (1.12-1.61)** | 1.15  (0.83-1.60) | 1.34  (0.94-1.89) | 1.18  (0.92-1.52) |
| **Lag = 9 months** | p-value | **<0.001** | 0.80 | 0.43 | 0.19 |
|  | HR  (95% CI) | **1.48  (1.24-1.76)** | 1.05  (0.74-1.48) | 1.18  (0.78-1.80) | 1.18  (0.92-1.50) |
| **Lag = 6 months** | p-value | **<0.001** | 0.12 | 0.12 | 0.36 |
|  | HR  (95% CI) | **1.48  (1.24-1.77)** | 1.31 (0.94-1.82) | 1.40  (0.92-2.13) | 1.12  (0.88-1.44) |
| **Lag = 3 months** | p-value | **<0.001** | 0.79 | 0.21 | 0.34 |
|  | HR  (95% CI) | **1.63  (1.37-1.94)** | 1.05  (0.73-1.53) | 1.35  (0.84-2.12) | 1.13  (0.88-1.45) |
| **Lag = 0 months** | p-value | **<0.001** | 0.67 | 0.06 | 0.08 |
|  | HR  (95% CI) | **1.61 (1.35-1.93)** | 1.08  (0.75-1.57) | 1.54  (0.98-2.42) | 1.25  (0.98-1.59) |
|  | Regimen | CNI+antiM | CNI+steroid | CNI+antiM+ steroid | mTOR inhibitor-based regimens |

**Table S5**: Covariate-adjusted hazard ratios (HR) for cumulative exposure (per 6-month increase)

| **Regimen** | **Adjusted HR (95% CI),**  **per 6-month increase** | **P value** |
| --- | --- | --- |
| CNI monotherapy | 1.13  (0.98,1.31) | 0.11 |
| CNI+antiM | 1.18  (1.02,1.37) | 0.027 |
| CNI+steroid | 1.14  (0.96,1.33) | 0.13 |
| CNI+antiM+steroid | 1.17  (0.99,1.38) | 0.07 |
| mTOR-based | 1.10  (0.95,1.28) | 0.21 |

**Table S6**: Element specific covariate-adjusted hazard ratios (HR) for cumulative exposure (per 6-month increase)

| **Element** | **Adjusted HR (95% CI)**  **per 6-month increase** | **P value** |
| --- | --- | --- |
| CNI | 1.13  (0.98,1.31) | 0.100 |
| antiM | 1.04  (1.01,1.08) | 0.005 |
| Steroid | 0.99  (0.95,1.06) | 0.96 |
